# Postictal Todd’s Paralysis Mimicking Acute Ischemic Stroke in Medullary Melanocytoma: A Multimodal Diagnostic Challenge

**DOI:** 10.7759/cureus.104991

**Published:** 2026-03-10

**Authors:** Sara Ali, Kian Memari, Lissette P Lazo, Shane Williams, Peter Cohen

**Affiliations:** 1 Medicine, Nova Southeastern University Dr. Kiran C. Patel College of Osteopathic Medicine, Clearwater, USA; 2 Family Medicine, Palmetto General Hospital, Hialeah, USA; 3 Family Medicine, Nova Southeastern University Dr. Kiran C. Patel College of Osteopathic Medicine, Fort Lauderdale, USA

**Keywords:** focal nonconvulsive seizure, medullary melanocytoma, postictal paralysis, stroke mimics, todd's paralysis

## Abstract

Acute focal neurologic deficits in patients with intracranial malignancy frequently prompt emergent evaluation for ischemic stroke. However, seizure-related deficits such as Todd’s paralysis may closely mimic cerebrovascular events. Todd’s paralysis consists of transient postictal neurological deficits that may persist from minutes to several days, particularly in patients with structural brain disease. We report a 45-year-old man with medullary melanocytoma who presented with acute left-sided weakness, facial droop, and dysarthria. CT perfusion demonstrated focal hypoperfusion suggestive of ischemic penumbra, and his National Institutes of Health Stroke Scale (NIHSS) score was 14. Diffusion-weighted MRI showed no evidence of acute infarction, and CT angiography demonstrated no large vessel occlusion. Electroencephalography revealed focal slowing over the left hemisphere without epileptiform discharges, consistent with a postictal state. His neurological deficits resolved within 24 hours. In the context of transient symptoms, absence of diffusion restriction, electrophysiologic findings, hyponatremia, and extensive structural brain disease, a diagnosis of focal nonconvulsive seizure with postictal Todd’s paralysis was favored. This case highlights the potential for perfusion imaging to mimic ischemia in neuro-oncology patients and underscores the importance of integrating multimodal imaging, metabolic evaluation, electrophysiologic data, and clinical evolution in acute stroke assessment.

## Introduction

Acute focal neurological deficits frequently trigger emergency stroke protocols. However, stroke mimics account for approximately 20-30% of acute stroke activations, with seizures representing one of the most common nonvascular causes [1,2]. Accurate differentiation is critical, as management strategies differ substantially.

Todd’s paralysis refers to transient focal neurological deficits following seizure activity and occurs in approximately 6-13% of patients after focal or generalized seizures [3]. These deficits may include unilateral weakness, speech disturbance, visual field deficits, or gaze deviation, often closely resembling acute ischemic stroke.

The underlying mechanism is thought to involve transient neuronal exhaustion and postictal hypoperfusion [3,4]. Advanced neuroimaging studies demonstrate that perfusion imaging during the postictal phase may show focal hypoperfusion that mimics ischemic penumbra, complicating interpretation [5]. Although diffusion-weighted MRI is highly sensitive for acute ischemia, very early or small infarcts may occasionally be diffusion-negative [6].

In patients with intracranial malignancy, diagnostic complexity increases. Medullary melanocytoma is a rare neuroectodermal tumor arising from leptomeningeal melanocytes and may predispose to tumor-associated epileptogenesis due to cortical disruption, inflammatory signaling, and vasogenic edema [7]. We present a case in which CT perfusion findings suggested ischemia, but multimodal evaluation supported a postictal mechanism.

## Case presentation

A 45-year-old man with medullary melanocytoma involving supratentorial and infratentorial regions presented with acute-onset left-sided weakness, facial droop, dysarthria, and impaired comprehension. His oncologic history included partial brain mass resection followed by chemotherapy, radiotherapy, and ongoing immunotherapy. Baseline functional status corresponded to a modified Rankin Scale (mRS) score of 3 [8].

Symptoms progressed over approximately one hour before presentation. On examination, he was responsive but unable to complete full phrases, although repetition was preserved. Cranial nerve examination revealed left facial weakness and horizontal right-beating nystagmus. Motor testing demonstrated left-sided weakness. Coordination testing showed dysmetria of the right upper and lower extremities. Sensory examination was limited due to poor cooperation.

**Figure 1:**
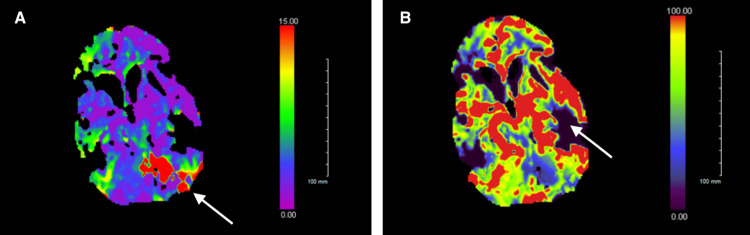
CT Cerebral Perfusion Scan (A) CT cerebral perfusion scan reveals ischemic penumbra around the superior aspect of the left cerebellum and left occipital lobe (white arrow). (B) CT cerebral perfusion scan illustrates small areas of acute infarction in the cortical to subcortical regions of the left precentral gyrus (white arrow).

Initial laboratory evaluation revealed mild leukocytosis and hyponatremia (sodium 125 mmol/L) (Table 1). CT perfusion demonstrated focal hypoperfusion involving the superior aspect of the left cerebellum and left occipital lobe, interpreted as suggestive of ischemic penumbra (Figure 1). The National Institutes of Health Stroke Scale (NIHSS) score was 14, reflecting deficits in language, facial palsy, motor strength, and coordination [9].

**Table 1:** Detailed Laboratory Investigations Abbreviations: µL: microliter, mm³: cubic millimeter, gm/dL: grams per deciliter, fL: femtoliter, pg: picogram, mmol/L: millimoles per liter, mg/dL: milligrams per deciliter, mcg/dL: micrograms per deciliter, U/L: units per liter, mL/min/1.73 m²: milliliters per minute per 1.73 square meters of body surface area,  WNL: within normal limits

Parameter (Unit)	Patient’s Value	Normal Reference Range	Clinical Note/Interpretation
White Blood Cell (µL)	13.4	5.0 - 11.0 x 10^3^	
Red Blood Cell (mm^3^)	4.16	4.70 - 6.10 x 10^6^	
Hemoglobin (gm/dL)	12.9	14.0 - 18.0	
Hematocrit (%)	36.1	42.0 - 52.0	
Mean Corpuscular Volume (fL)	87	80 - 94	Within Normal Limits (WNL)
Mean Corpuscular Hemoglobin (pg)	31.0	27.0 - 31.0	WNL
Mean Corpuscular Hemoglobin Concentration (gm/dL)	35.7	33.0 - 37.0	WNL
Red Cell Distribution Width - Coefficient of Variation (%)	13.3	11.5 - 14.5	WNL
Platelet Count (µL)	323	130 - 140 x 10^3^	WNL
Sodium (mmol/L)	125	137 - 145	
Potassium (mmol/L)	4.0	3.4 - 5.0	WNL
Chloride (mmol/L)	90	98 - 107	
Carbon Dioxide (mmol/L)	21	22 - 30	
Blood Urea Nitrogen (mg/dL)	9	9.0 - 20.0	
Creatinine (mg/dL)	0.80	0.66 - 1.25	WNL
Estimated Glomerular Filtration Rate (mL/min/1.73 m^2^)	111	≥ 90	WNL
Glucose (mg/dL)	193	74.0 - 106.0	
Calcium (mg/dL)	8.5	8.4 - 10.2	WNL
Phosphorus (mg/dL)	2.9	2.5 - 4.5	WNL
Total Bilirubin (mcg/dL)	0.60	0.20 - 1.30	WNL
Aspartate Aminotransferase (U/L)	22	17 - 59	WNL
Alanine Aminotransferase (U/L)	11	21 - 72	
Alkaline Phosphatase (U/L)	64	38 - 126	WNL
Total Creatine Kinase (U/L)	34	55 - 170	
Cortisol AM Sample (mcg/dL)	55.20	10.00 - 30.00	
Urine Color	Straw	Yellow	WNL
Urine Clarity	Clear	Clear	WNL
Urine pH	7.0	5.0 - 8.0	WNL
Urine Specific Gravity	1.058	1.005 - 1.030	
Urine Protein	Negative	Negative	WNL
Urine Glucose (mg/dL)	Negative	Negative	WNL
Urine Nitrate	Negative	Negative	WNL
Urine Bilirubin	Negative	Negative	WNL
Urine Leukocyte Esterase	Negative	Negative	WNL
Urine Toxicology Screen	Negative	Negative	WNL

**Figure 2:**
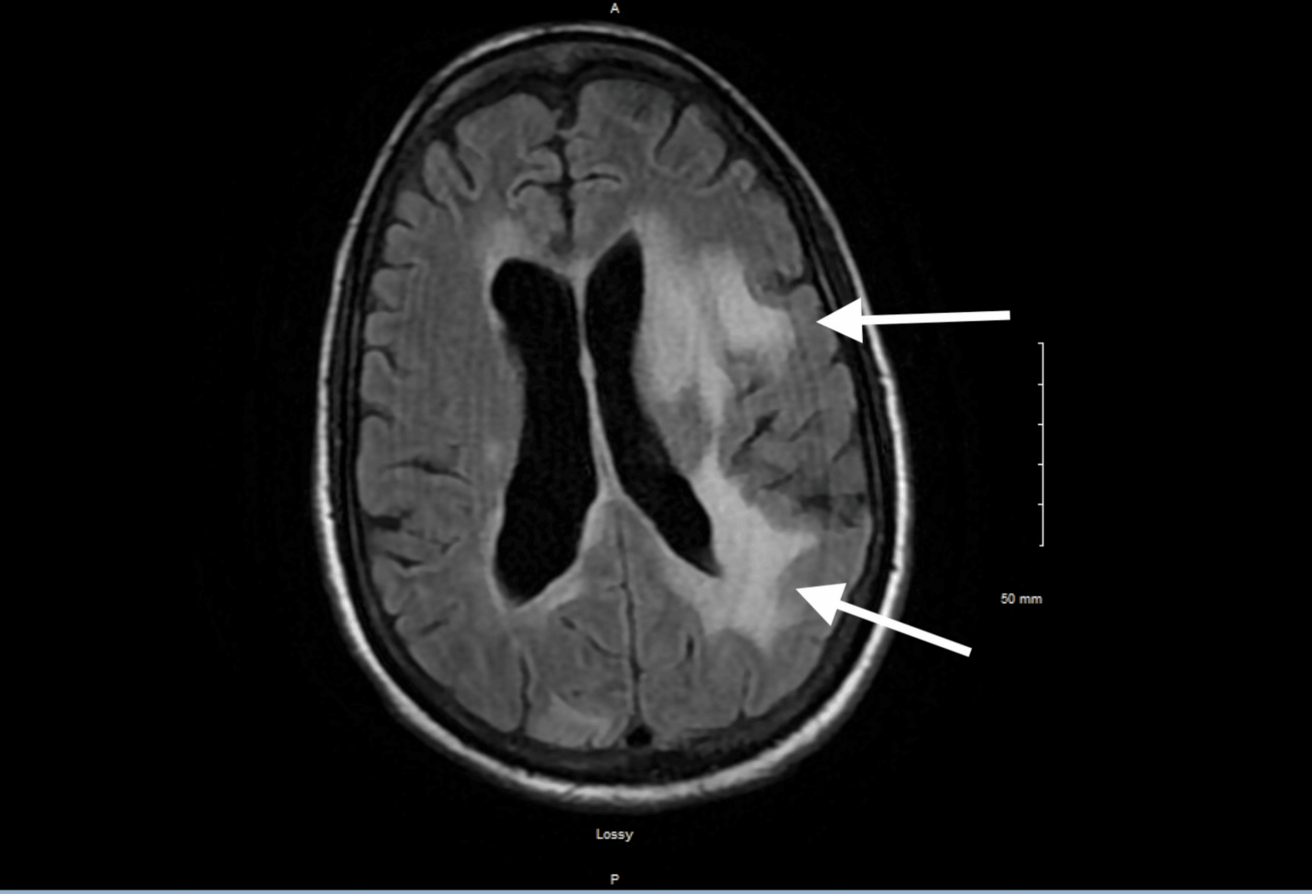
Contrast-Enhanced MRI of the Brain MRI of the brain with contrast shows multiple enhancing lesions in the supratentorial brain and left cerebellum (white arrows), predominantly in periventricular distribution.

MRI of the brain with diffusion-weighted imaging demonstrated multiple enhancing lesions with vasogenic edema, indicating a perfusion abnormality consistent with a stroke mimic in context (Figure 2). CT angiography revealed no evidence of large vessel occlusion, stenosis, or dissection.

Within 24 hours, neurological deficits resolved completely. Electroencephalography (EEG) demonstrated focal slowing over the left hemisphere without epileptiform discharges, consistent with a postictal state.

Given the transient course, absence of diffusion restriction, EEG findings, structural brain disease, and metabolic derangement, a diagnosis of focal nonconvulsive seizure with postictal Todd’s paralysis was favored. The patient was initiated on levetiracetam and continued on dexamethasone for cerebral edema. He was transferred for continued neuro-oncologic management.

## Discussion

This case illustrates the diagnostic challenge of distinguishing postictal deficits from acute ischemic stroke in patients with intracranial disease. The patient’s abrupt focal deficits and CT perfusion abnormalities initially raised concern for ischemia.

Seizure-related perfusion abnormalities are well documented. During ictal phases, focal hyperperfusion may occur, whereas postictal states are frequently associated with focal hypoperfusion that can resemble ischemic penumbra on CT perfusion imaging [5,10]. In this case, perfusion findings were initially concerning but were not accompanied by diffusion restriction on MRI.

Although CT angiography excluded large vessel occlusion, small-vessel ischemia cannot be ruled out solely by negative vascular imaging. Furthermore, diffusion-weighted MRI may be falsely negative in very early or small infarcts [6]. Therefore, imaging findings must be interpreted in conjunction with clinical progression.

The patient’s rapid neurological recovery and EEG findings of focal hemispheric slowing supported a postictal mechanism. Focal slowing without epileptiform discharges is commonly observed following seizure activity and reflects transient cortical dysfunction.

Experimental models suggest postictal hypoperfusion may persist for approximately one hour [4]; however, clinical Todd’s paralysis may last from minutes to several days, particularly in patients with underlying structural lesions. In this case, extensive intracranial disease and vasogenic edema likely prolonged neuronal recovery.

Hyponatremia may have contributed to seizure susceptibility by lowering neuronal depolarization thresholds and enhancing cortical excitability. Electrolyte disturbances are recognized precipitants of seizure activity and should be carefully evaluated in similar presentations.

This case emphasizes the importance of multimodal evaluation in neuro-oncology patients presenting with acute focal deficits. Reliance on perfusion imaging alone may lead to diagnostic uncertainty. Integration of clinical trajectory, diffusion-weighted MRI, vascular imaging, metabolic evaluation, and electrophysiologic data is essential.

## Conclusions

Postictal Todd’s paralysis should be considered in neuro-oncology patients presenting with acute focal neurological deficits and perfusion abnormalities suggestive of ischemia. In this case, absence of diffusion restriction, rapid symptom resolution, and EEG findings of focal hemispheric slowing supported a postictal mechanism rather than acute infarction. Multimodal diagnostic assessment is critical to avoid misclassification and ensure appropriate management in complex clinical settings.
